# ILPMDA: Predicting miRNA–Disease Association Based on Improved Label Propagation

**DOI:** 10.3389/fgene.2021.743665

**Published:** 2021-09-30

**Authors:** Yu-Tian Wang, Lei Li, Cun-Mei Ji, Chun-Hou Zheng, Jian-Cheng Ni

**Affiliations:** ^1^School of Cyber Science and Engineering, Qufu Normal University, Qufu, China; ^2^School of Artificial Intelligence, Anhui University, Hefei, China

**Keywords:** miRNA, disease, similarity kernel fusion, improved label propagation, miRNA–disease association

## Abstract

MicroRNAs (miRNAs) are small non-coding RNAs that have been demonstrated to be related to numerous complex human diseases. Considerable studies have suggested that miRNAs affect many complicated bioprocesses. Hence, the investigation of disease-related miRNAs by utilizing computational methods is warranted. In this study, we presented an improved label propagation for miRNA–disease association prediction (ILPMDA) method to observe disease-related miRNAs. First, we utilized similarity kernel fusion to integrate different types of biological information for generating miRNA and disease similarity networks. Second, we applied the weighted k-nearest known neighbor algorithm to update verified miRNA–disease association data. Third, we utilized improved label propagation in disease and miRNA similarity networks to make association prediction. Furthermore, we obtained final prediction scores by adopting an average ensemble method to integrate the two kinds of prediction results. To evaluate the prediction performance of ILPMDA, two types of cross-validation methods and case studies on three significant human diseases were implemented to determine the accuracy and effectiveness of ILPMDA. All results demonstrated that ILPMDA had the ability to discover potential miRNA–disease associations.

## Introduction

MicroRNAs (miRNAs) are a class of short non-coding RNA (∼22 nt) molecules encoded by endogenous genes (Ambros, 2001; Bartel, 2004; He and Hannon, 2004; Ribeiro et al., 2014). Since their initial discovery 20 years ago, many miRNAs have been revealed (Wightman et al., 1993; Jopling et al., 2005; Ana and Sam, 2014). Increasing numbers of miRNAs are confirmed to play important roles in critical biological processes (Meola et al., 2009), such as cell growth, proliferation, metabolism, apoptosis, and signal transduction (Xu et al., 2004; Cheng et al., 2005; Karp and Ambros, 2005; Miska, 2005; Alshalalfa and Alhajj, 2013). Studies have shown that the emergence and development of various human diseases are closely related to miRNAs (Samira et al., 2013; Yanaihara et al., 2013). Importantly, disease-related miRNAs are regarded as potential biomarkers that could significantly contribute to understanding the mechanisms of various complex human diseases and enable their prevention, detection, diagnosis, and treatment (Lynam-Lennon et al., 2009; Meola et al., 2009; Ye et al., 2016). Numerous traditional experiments have been conducted to predict the unknown relationship between miRNAs and diseases, but only few miRNA–disease associations have been confirmed (Thomson et al., 2007; Han et al., 2014). In addition, traditional methods are generally time-consuming and expensive. In order to overcome the shortcomings of traditional methods, considerable computational models have been proposed to predict disease-related miRNAs. These computational models could obtain more accurate prediction results, which may make future development in the field of biological research more convenient.

In the past few years, several prediction models have been proposed based on the theory that functionally similar miRNAs tend to be related to phenotypically similar diseases, and vice versa (Zeng et al., 2016; You et al., 2017). Jiang et al. (2010) established a new computation-based model that identified potential miRNA–disease connections by employing the hypergeometric distribution; however, the similarity information applied in this model excluded similarity scores. To predict possible disease-related miRNAs, Li et al. (2011) constructed a novel model that employed a functional consistency score between target and disease genes. Søren et al. (2014) constructed an miPRD model to infer miRNA–protein and disease–protein connections. These connections were then exploited to predict the relationship between miRNAs and diseases. In addition, a new framework named ranking-based k-nearest neighbor for miRNA–disease association prediction (RKNNMDA) employed the k-nearest neighbor (KNN) algorithm to obtain the neighbors of miRNAs and diseases (Chen et al., 2016). RKNNMDA also involved the support vector machine (SVM) ranking model to obtain the ranking results of the KNNs. Then, RKNNMDA implemented weighted voting on the ranking results to obtain all possible miRNA–disease connections. Moreover, this model could demonstrate possible unverified connections between miRNAs and diseases. The Jaccard similarity between miRNAs and diseases was introduced by Chen et al. (2018a) in the bipartite local models and hubness-aware regression for miRNA–disease association prediction (BLHARMDA) model, which also took advantage of a bipartite local model with a KNN framework for improving the prediction efficiency. Chen et al. (2019a) proposed a computational framework, MDVSI, to predict potential miRNA–disease connections by integrating different miRNA similarities (miRNA functional and topological similarity). MDVSI employed the linear weight method to integrate miRNA similarities and then used the recommendation method to infer unknown relationships between miRNAs and diseases. Furthermore, Zhou et al. (2020) utilized gradient boosting decision tree and logistic regression analysis to predict disease-associated miRNAs. The gradient boosting decision tree method was used to extract miRNA and disease features; then, the logistic regression method used these new features to obtain a final miRNA–disease association score.

As artificial intelligence technology has developed, machine learning-based models have been increasingly employed to predict the accuracy of miRNA–disease associations. Chen and Yan (2014) adopted semi-supervised learning in the regularized least squares for miRNA–disease association (RLSMDA) model to efficiently predict feasible miRNA–disease relationships. RLSMDA could calculate accuracy correlation scores between miRNAs and diseases, with the advantage that the model could avoid using negative samples. Xu et al. (2014) acquired disease-associated miRNAs by the miRNA target-dysregulated network (MTDN) model. To achieve more accurate prediction results, they utilized the feature obtained by network topology information and an SVM classifier to identify positive miRNA–disease connections from negative samples; however, negative samples were difficult to obtain. Chen et al. (2015) first employed the restricted Boltzmann machine for multiple types of miRNA–disease association prediction (RBMMMDA) models to make predictions. The miRNA–disease relationships in RBMMMDA were represented by a two-layer undirected graph that contained a visible and hidden layer. The advantage of this model was its ability to facilitate understanding of the mechanisms of diseases according to the miRNAs. Li et al. (2017) sought to avoid utilizing negative samples so as to achieve accurate prediction results in matrix completion for miRNA–disease association prediction (MCMDA). The model could employ validated miRNA–disease connections to determine unknown connections. Furthermore, Chen et al. (2017a) utilized ensemble learning and link prediction to infer feasible miRNA–disease relationships. Based on global similarity measures, ranking results that were obtained from three traditional methods of similarity measurement were integrated by the ensemble learning method to improve the accuracy of the prediction results. The probabilistic matrix factorization (PMF) algorithm was used to infer unknown miRNA–disease interactions (Xu et al., 2019). The PMF algorithm is a machine learning technique that is widely used in recommender systems; thus, it could effectively apply all information to recommend miRNAs that are related to the disease in question. Peng et al. (2019) proposed the miRNA–disease association-convolutional neural network (MDA-CNN) model for identifying miRNA–disease interactions. The miRNA–disease interaction features were first captured by a three-layer network. Then, an autoencoder was employed to identify obvious miRNA–disease feature combinations. After these feature representations were reduced, a CNN was employed to obtain the ultimate prediction performance. Li et al. (2020) proposed a new method of neural inductive matrix completion with graph convolutional network, named NIMCGCN, to predict potential miRNA–disease associations. The miRNA and disease latent feature representations were extracted from the miRNA and disease similarity networks by graph convolutional networks in NIMCGCN. Then, the learned latent feature representations were input into the neural inductive matrix completion model to complete the missing miRNA–disease associations. Guo et al. (2021) presented a novel model, MLPMDA, which implemented multilayer linear projection to predict miRNA–disease interactions. They utilized the top nearest neighbors of entities to process miRNA–disease interaction information; the updated miRNA–disease interaction and disease similarity constituted a heterogeneous matrix. The multilayer projection and layer stacking strategy were used on the heterogeneous matrix to make predictions. However, MLPMDA requires high-quality biological data to achieve reliable and stable performance. A novel method of neural multiple-category miRNA–disease association prediction named NMCMDA was proposed by Wang et al. (2021) to observe the unknown disease-related miRNAs. The two main components in NMCMDA were encoder and decoder. The encoder was implemented on the heterogeneous network of miRNA–disease and used graph neural network to extract miRNA and disease latent features. The decoder applied these latent features to obtain miRNA–disease association scores. Different kinds of encoders and decoders were put forward for NMCMDA. Ultimately, the combination of relational graph convolutional network encoder and neural multirelational decoder in NMCMDA reached the best prediction performance. Huang et al. (2021) presented a new tensor decomposition-based model, named TDRC, which integrates the data of miRNA–miRNA similarity and disease–disease similarity as decomposition constraints. Experimental results demonstrated that TDRC further improved prediction performance by comparing it with previous tensor decomposition models. Zhang et al. (2021) presented a novel method of fast linear neighborhood similarity-based network link inference (FLNSNLI) to predict unverified associations of miRNA–disease. FLNSNLI first formulated the verified miRNA–disease associations as a bipartite network and expressed miRNAs (diseases) as association profile. Then, association profiles and fast linear neighborhood similarity measure were used to calculate miRNA–miRNA similarity and disease–disease similarity. Furthermore, FLNSNLI implemented label propagation method to score candidate miRNA–disease associations based on miRNA–miRNA similarity and disease–disease similarity, respectively. The two results were integrated by the weighted average strategy to observe unknown miRNA–disease associations.

In this study, we presented a novel model named improved label propagation for miRNA–disease association prediction (ILPMDA) to infer potential associations between miRNAs and diseases. We utilized SKF to fuse different disease similarity matrices (disease semantic similarity, disease functional similarity, and disease Gaussian interaction profile kernel similarity) and miRNA similarity matrices (miRNA functional similarity, miRNA sequence similarity, and miRNA Gaussian interaction profile kernel similarity) for generating reliable disease and miRNA similarity networks. We also used WKNKN to update the unknown miRNA–disease association matrix to reduce its sparsity. Improved label propagation was then conducted on two types of similarity networks to predict the miRNA–disease association scores. We integrated these two correlation score matrices to obtain the final prediction results. The global leave-one-out cross-validation (LOOCV) and fivefold cross-validation (5-CV) were used to evaluate our model. Consequently, ILPMDA individually achieved area under the receiver operating characteristic (ROC) curve (AUC) values of 0.9751 and 0.9501 for LOOCV and 5-CV, respectively. Furthermore, two kinds of case studies on colon neoplasms, prostate neoplasms, and breast neoplasms further demonstrated that ILPMDA could be an effective method for discovering unverified miRNA–disease associations.

## Materials and Methods

### Human miRNA–Disease Associations

**FIGURE 1:**
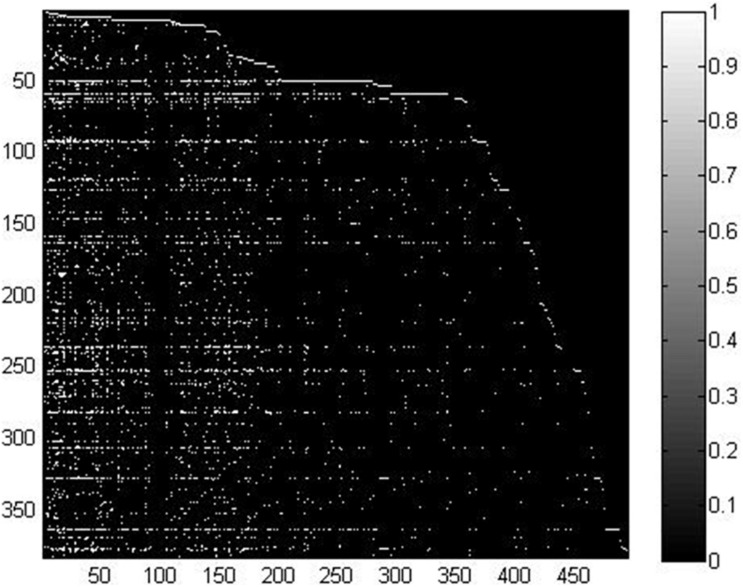
Visualization of the miRNA–disease association matrix.

In this study, we took advantage of miRNA–disease association data from the HMDD v2.0 database (Yang et al., 2013), which contained 5,430 verified associations between 495 miRNAs and 383 diseases. For convenient calculation, we constructed an adjacency matrix *A* ∈ *R*^*nd*×*nm*^ to represent the miRNA–disease relationship. We set *nd* and *nm* to represent the number of diseases and miRNAs, respectively. Specifically, the element *A*(*i*, *j*) is equal to 1 when disease *d*_*i*_ is shown to be connected with miRNA *m*_*j*_; otherwise, it is set to 0. In order to clearly demonstrate the detailed information of matrix *A*, we visualized it in Figure 1. We used white points and black points to denote known associations and unknown associations, respectively. According to the distribution of points in Figure 1, number of known associations is far less than the number of unknown associations, which means the matrix *A* can be considered a sparse matrix.

### Disease Semantic and Functional Similarity

Based on the theory proposed by Wang et al. (2010), disease similarity can be calculated using semantic information. We used *SD*_1_ ∈ *R*^*nd*×*nd*^ to denote disease semantic similarity, which could be obtained by utilizing the disease arborescence attribute in Mesh database (Lowe and Barnett, 1994). In this database, disease nodes were labeled in a directed acyclic graph (DAG). Diseases that relate to the same genes are likely to have similar phenotypes; as such, disease functional similarity is calculated by the disease–gene connections (Luo et al., 2017; Jiang L. et al., 2018). In addition, we adopted disease functional similarity information from previous literature (Jiang L. et al., 2018) and utilized *SD*_2_ ∈ *R*^*nd*×*nd*^ to denote disease functional similarity. The element *SD*_2_(*d*_*i*_, *d*_*j*_) represents the value of similarity between disease *d*_*i*_ and disease *d*_*j*_.

### miRNA Functional and Sequence Similarity

MicroRNAs with similar functions have a high probability of being related to diseases that are similar, and vice versa (Lu et al., 2008) (Goh, Cusick et al., Wang, Zaman et al., Lu, Zhang et al. 2008) (Goh, Cusick et al., Sanghamitra, Bandyopadhyay et al., Lu, Zhang et al. 2008) (Goh, Cusick et al., Lu, Zhang et al. 2008, Bandyopadhyay, Mitra et al. 2010)[38-40]. Therefore, we downloaded the miRNA function similarity information from http://www.cuilab.cn/files/images/cuilab/misim.zip (Wang et al., 2010). The miRNA sequence similarity information was acquired from the miRBase database (Kozomara and Griffiths-jones, 2013). For convenient and efficient calculation, we constructed the matrix *SM*_1_ ∈ *R*^*nm*×*nm*^ and *SM*_2_ ∈ *R*^*nm*×*nm*^ to store the miRNA functional similarity and sequence similarity data, respectively.

### Gaussian Interaction Profile Kernel Similarity for Diseases and miRNAs

Gaussian interaction profile (GIP) kernel similarity was used to represent miRNA and disease similarity (Van et al., 2011; Chen et al., 2017b). First, we denoted vector *K*(*d*_*i*_) to represent the interaction profile of disease *d*_*i*_ in accordance with whether *d*_*i*_ had a verified association with each miRNA. Similarly, we denoted vector *K*(*m*_*i*_) to represent the interaction profile *m*_*i*_ in accordance with whether *m*_*i*_ had a verified association with each disease. The equation to calculate GIP kernel similarity for diseases is as follows:


(1) $$S\,D_{3}\,\left(d_{i},d_{j}\right)=e\,x\,p\,\left(-\rho_{d}\,{∥K\,\left(d_{i}\right)-K\,\left(d_{j}\right)∥}^{2}\right)\;$$

where *SD*_3_(*d*_*i*_, *d*_*j*_) indicates the GIP kernel similarity between disease *d*_*i*_ and disease *d*_*j*_, ρ_*d*_ is applied to control kernel bandwidth. ρ_*d*_ is obtained by normalizing the original bandwidth $\rho_{d}^{\prime }$ to the average number of verified associations with miRNAs per disease, as follows:


(2) $$\rho_{d}=\rho_{d}^{\prime }/\left(\frac{1}{n\,d}\,\sum_{i=1}^{n\,d}{||\left|K\,\left(d_{i}\right)\right|||}^{2}\right)$$

Similarly, the equations to calculate GIP kernel similarity for miRNAs are as follows:


(3) $$S\,M_{3}\,\left(m_{i},m_{j}\right)=e\,x\,p\,\left(-\rho_{m}\,{∥K\,\left(m_{i}\right)-K\,\left(m_{j}\right)∥}^{2}\right)$$

(4) $$\rho_{m}=\rho_{m}^{\prime }/\left(\frac{1}{n\,m}\,\sum_{i=1}^{n\,m}{∥\left|K\,\left(m_{i}\right)\right|∥}^{2}\right)$$

where *SM*_3_(*m*_*i*_, *m*_*j*_) indicates the GIP kernel similarity between miRNA *m*_*i*_ and miRNA *m*_*j*_; ρ_*m*_ is also employed to control kernel bandwidth.

### Similarity Kernel Fusion

**FIGURE 2:**
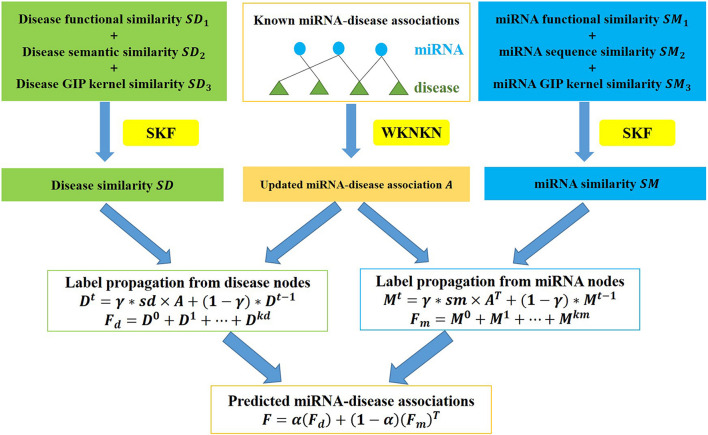
Flow chart of ILPMDA to predict unknown associations based on the known associations in the HMDD v2.0 database.

A flow chart of ILPMDA is shown in Figure 2. After we obtained disease semantic similarity, disease functional similarity, and disease GIP kernel similarity, the similarity kernel fusion (Jiang L. et al., 2018; Jiang et al., 2019) was implemented to integrate them into ultimate disease similarity. Similarly, miRNA functional similarity, miRNA sequence similarity, and miRNA GIP kernel similarity were integrated into ultimate miRNA similarity by implementing the SKF method. The specific integration process of disease similarity matrices is described in the following discussion.

First, three different disease similarities were treated as original disease similarity kernels, which were defined as *SD*_*n*_, *n* = 1, 2, 3 in the above sections. The similarity of each disease was normalized using the following equation:


(5) $$P_{n}\,\left(d_{i},d_{j}\right)=\frac{S\,D_{n}\,\left(d_{i},d_{j}\right)}{\sum_{d_{k}\in D}S\,D_{n}\,\left(d_{k},d_{j}\right)}$$

where *P*_*n*_ indicates the normalized kernel that satisfies the condition of ∑_*dk*∈*D*_ *P*_*n*_(*d*_*i*_, *d*_*j*_) = 1, and $D={\left\{d_{i}\right\}}_{i=1}^{n\,d}$ indicates the set of diseases.

Second, the neighbor-constraint kernel for each original disease kernel was constructed by the following equation:


(6) $$\begin{array}{c} C_{n}\,\left(d_{i},d_{j}\right)=\left\{ \begin{array}{cc} \frac{S\,D_{n}\,\left(d_{i},d_{j}\right)}{\sum_{d_{k}\in N_{i}}S\,D_{n}\,\left(d_{i},d_{k}\right)}\; & \text{if}\,d_{j}\in N_{i}\\ 0\; & \text{if}\,d_{j}\notin N_{i} \end{array}\right. \end{array}$$

where *C*_*n*_ (*d*_*i*_, *d*_*j*_) denotes a neighbor-constraint kernel that satisfies the condition of ∑_*dk*∈*D*_ *C*_*n*_(*d*_*i*_, *d*_*j*_) = 1 and *N_i_* denotes the collection of all neighbors of disease *d*_*i*_, including itself.

Third, the normalized kernels and neighbor-constraint kernels were integrated as follows:


(7) $$P_{n}^{l+1}=\beta \,\left(C_{n}\times \frac{\sum_{t\neq n}P_{t}^{l}}{2}\times C_{n}^{T}\right)+\left(1-\beta \right)\,\frac{\sum_{t\neq n}P_{t}^{0}}{2}$$

where $P_{n}^{l+1}$ represents the value of the *n*th kernel after *l+1* iterations, $P_{t}^{0}$ represents the initial value of *P_t_*, and the weight parameter β ∈ (0, 1) is used to balance the rate. After $P_{n}^{l+1}$, *n* = 1, 2, 3 is obtained, the overall kernel *SD*^∗^ can be calculated by the following formula:


(8) $$S\,D^{*}=\frac{1}{3}\,\sum_{n=1}^{3}P_{n}^{l+1}$$

Fourth, a weighted matrix *W* is applied to further eliminate noise in the overall kernel *SD*^∗^. The process for constructing *W* is as follows:


(9) $$\begin{array}{c} W\,\left(d_{i},d_{j}\right)=\left\{ \begin{array}{cc} 1\; & \text{if}\,d_{i}\in N_{j}\cap d_{j}\in N_{i}\\ 0\; & \text{if}\,d_{i}\notin N_{j}\cap d_{j}\notin N_{i}\\ 0.5\; & \text{otherwise} \end{array}\right. \end{array}$$

Last, the final disease similarity kernel *SD* ∈ *R*^*nd*×*nd*^ can be computed as follows:


(10) $$S\,D=W\times S\,D^{*}$$

Similarly, we could obtain the final miRNA similarity kernel as *SM* ∈ *R*^*nm*×*nm*^.

### Weighted k-Nearest Known Neighbors

In order to make the experimental data more accurate and improve the prediction accuracy, we applied the method of weighted k-nearest known neighbor (Ezzat et al., 2017) to the adjacency matrix *A* ∈ *R*^*nm*×*nd*^. *A*(*d*_*i*_) = (*A*_*i*1_, *A*_*i*2_,, *A*_*ind*_) and *A*(*m*_*j*_) = (*A*_1*j*_, *A*_2*j*_,, *A*_*nmj*_) indicate the interaction profiles for *d_i_* and *m_j_*, respectively. The procedure for using the WKNKN algorithm included several steps.

First, the similarity between each disease and its k-nearest verified diseases was employed to construct the interaction profile. For example, the k interaction profiles between miRNA *d*_*r*_ and its KNNs are represented by *AD*(*d*_*r*_), which is obtained by the following formula:


(11) $$A\,D\,\left(d_{r}\right)=\frac{1}{N_{d}}\,\sum_{i=1}^{K}\rho_{i}\,A\,\left(d_{i}\right)$$

where *N*_*d*_ = ∑_1≤__*i*__≤__*K*_ *SD*(*d*_*i*_, *d*_*j*_) is the normalization term. The weight coefficient ρ_*i*_ = *t*^*i*−1^*SD (*d*_*i*_, *d*_*r*_) is employed to control the similarity between *d_i_* and *d*_*r*_, where 0≤ *t* ≤ 1 is the corresponding balance parameter. Similarly, the k interaction profiles between disease *m*_*r*_ and its KNNs are represented by *AM* (*m*_*r*_), which can be obtained as follows:


(12) $$A\,M\,\left(m_{r}\right)=\frac{1}{N_{m}}\,\sum_{j=1}^{K}\rho_{j}\,A\,\left(m_{j}\right)$$

Second, the *AD* and *AM* are combined to obtain the matrix *A*_*md*_, which indicates the new miRNA–disease interaction profile. The specific process is depicted by the following formula:


(13) $$A_{m\,d}=\left(\delta_{1}AD+\delta_{2}AM\right)/\sum \delta_{i}\left(i=1,2\right)$$

Last, the matrix *A*_*md*_ is employed to update the original matrix *A*, and the corresponding formula is displayed as follows:


(14) $$A=m\,a\,x\,\left(A,A_{m\,d}\right)$$

### Improved Label Propagation

When the label propagation (Zhu and Ghahramani, 2002) method was implemented for the disease similarity network *SD*, we applied *A*(*i*,:) to represent the initial label of disease node *d_i_*, where *A*(*i*,:) denotes the *i*th row of the miRNA–disease association matrix *A*. In addition, this label information is propagated among neighboring nodes in similarity network *SD*. Thereafter, the label information of each disease node can be updated depending on the label information accepted from the neighboring nodes. However, according to the assumption that local neighboring nodes with high similarity scores are more reliable than remote neighboring nodes with low similarity scores, we employed the KNN algorithm to sort KNNs for each disease node. Hence, *Q*_*i*_ was utilized to denote the nearest neighboring nodes set of disease node *d*_*i*_. Then, its local affinity could be calculated as follows:


(15) $$\begin{array}{c} s\,d\,\left(d_{i},d_{j}\right)=\left\{ \begin{array}{cc} \frac{S\,D\,\left(d_{i},d_{j}\right)}{\sum_{d_{k}\in Q_{i}}S\,D\,\left(d_{i},d_{k}\right)}\; & \mathrm{if}\,d_{j}\in Q_{i}\\ 0\; & \text{otherwise} \end{array}\right. \end{array}$$

where *sd* denotes the local affinity matrix of the disease. Similarly, we could obtain the miRNA local affinity matrix*sm*. Thereafter, we constructed novel disease and miRNA weighted similarity networks that are more suitable for implementing the label propagation algorithm.

After obtaining the disease similarity matrix *sd* ∈ *R*^*nd*×*nd*^, miRNA similarity matrix *sm* ∈ *R*^*nm*×*nm*^, and the miRNA–disease association matrix *A* ∈ *R*^*nd*×*nm*^, we applied the bidirectional label propagation algorithm. The implantation of directional label propagation can be divided into three major steps.

In the first step, updating the label information of a specific disease *d*_*i*_ is affected by two parts of the labels. This involves absorbing labels from neighboring nodes and retaining previous labels. $D_{i}^{t}$ is employed to represent the label of disease *d*_*i*_ after *t* rounds of updating; then, $D_{i}^{t}$ can be calculated using the following equation:


(16) $$D_{i}^{t}=\gamma^{*}\,s\,d\,\left(d_{i},:\right)\times A+{\left(1-\gamma \right)}^{*}\,D_{i}^{t-1}$$

where γ ∈ [0, 1] is employed to balance the rate between absorbing labels from neighboring nodes and retaining previous labels, and $D_{i}^{0}=A\,\left(i,:\right)$ represents the initial label information of disease *d*_*i*_.

For all disease nodes, we could obtain their label vectors $D_{1}^{t}$, $D_{2}^{t}$, …, $D_{nd}^{t}$ after *t* rounds until convergence. We constructed the formula $D^{t}=(D_{1}^{t}$; $D_{2}^{t}$; …; $D_{nd}^{t}$, and Equation (16) could be rewritten as follows:


(17) $$D^{t}=\gamma^{*}\,s\,d\times A+{\left(1-\gamma \right)}^{*}\,D^{t-1}$$

The iteration of the above equation can be considered convergent. As such, when the difference between the last label matrix *D*^*t*^ and the former label matrix *D*^*t*−1^ is lower than the predetermined threshold, the process of iterative updating will stop. Hence, we assumed that the iterative process stopped after *kd* rounds of iterative updating. One miRNA–disease association score matrix *F_d_* could be obtained by the below formula:


(18) $$F_{d}=D^{0}+D^{1}+\ldots +D^{k\,d}$$

In the second step, for a random miRNA *m*_*i*_, the process of updating its label information is also affected by absorbing labels from neighbors with γ probability and remaining previous labels with 1−γ probability. $M_{i}^{t}$ is used to represent the label of miRNA *m*_*i*_ after *t* rounds of updating and $M_{i}^{0}=A\,\left(:,i\right)$ denotes the initial label information of miRNA *m_i_*. Then, $M_{i}^{t}$ can be calculated using the following formula:


(19) $$M_{i}^{t}=\gamma *s\,m\,\left(m_{i},:\right)\times A^{T}+\left(1-\gamma \right)*M_{i}^{t-1}$$

In all the miRNA nodes, we could obtain their label vectors $M_{1}^{t}$, $M_{2}^{t}$, …, $M_{nd}^{t}$ after *t* rounds until convergence. We constructed the formula $M^{t}=(M_{1}^{t}$; $M_{2}^{t}$; …; $M_{nm}^{t}$, and Equation (19) could be rewritten as follows:


(20) $$M^{t}=\gamma *s\,m\times A^{T}+\left(1-\gamma \right)*M^{t-1}$$

The iteration of the above equation can be considered convergent. Therefore, when the difference between last label matrix *M*^*t*^ and the former label matrix *M*^*t*−1^ is lower than the predetermined threshold, the process of iterative updating will stop. After *km* rounds of iterations, we could obtain another miRNA–disease association score matrix:


(21) $$F_{m}=M^{0}+M^{1}+\ldots +M^{k\,m}$$

In the third step, for the purpose of generating accuracy prediction results, the score matrices *F*_*m*_ and *F*_*d*_ can be integrated by an average ensemble method to obtain a final miRNA–disease association score matrix, as follows:


(22) $$F=\alpha \,\left(F_{d}\right)+\left(1-\alpha \right)\,{\left(F_{m}\right)}^{T}$$

where hyper-parameter α is set to 0.5, which is employed to balance the score matrices *F*_*d*_ and *F*_*m*_. The adjacency matrix *F* contains the predictive association score between miRNAs and diseases.

## Results

### Performance Evaluation

We utilized global LOOCV and 5-CV to evaluate the prediction performance of ILPMDA. As cross-validation is currently general practice in predicting miRNA/circular RNA (circRNA)/long noncoding RNA (lncRNA)–disease associations, this approach was selected for evaluation (Shen et al., 2017; Li et al., 2019; Wang et al., 2020). In the global LOOCV method, each verified association in the HMDD v2.0 database served as a test sample; the rest of the verified associations served as training samples, and the unknown associations were regarded as candidate samples. Similarly, in the 5-CV method, all verified associations in the HMDD v2.0 database were randomly divided into five parts in a random way; one group was treated as test samples, while the others were treated as training samples. The candidate is composed of unknown miRNA–disease associations. We applied repeated segmentations on verified positive samples multiple times to reduce potential deviations. We then implemented the model to calculate the score list of all associations and rank the candidate sample scores with the sample score in both LOOCV and 5-CV. If the ranking of the test sample was higher than the given threshold, the model was regarded as a successful prediction model. In addition, we could draw the ROC curve by employing the true-positive rate (TPR, sensitivity) against the false-positive rate (FPR, 1 − specificity). Sensitivity refers to the percentage of test samples with values higher than the threshold, and specificity refers to the percentage of negative associations with values lower than the threshold. The equations used to calculate FPR and TPR are demonstrated as follows:


(23) $$F\,P\,R=\frac{F\,P}{T\,N+F\,P}$$

(24) $$T\,P\,R=\frac{T\,P}{T\,P+F\,N}$$

where *FP* indicates the quantity of samples that are negative samples but are considered as positive samples, *TP* denotes the quantity of samples that are positive samples and are considered as positive samples, and *FN* and *TN* are identified as the opposite of *FP* and *TP*, respectively. Hence, we could utilize the AUC values between 0 and 1 as evaluative criteria; a greater AUC indicated that the model had better prediction performance. Simulation results showed that ILPMDA achieved AUCs of 0.9751 and 0.9501 for the global LOOCV and 5-CV methods, respectively, demonstrating that ILPMDA achieved excellent prediction performance.

**FIGURE 3:**
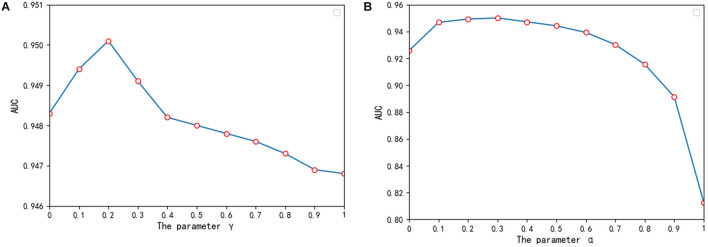
The influence of different parameters on ILPMDA. **(A)** The influence of parameter γ. **(B)** The influence of parameter α.

We also optimized two significant parameters γ and α in the ILPMDA model, which were utilized to balance the rate of absorbing labels from neighboring nodes and retaining previous labels and the rate between the score matrix *F*_*d*_ and score matrix *F*_*m*_. In order to select the best value of parameter γ, we applied the AUCs of 5-CV to evaluate γ ∈ {0, 0.1, 0.2,,1}. According to the evaluation results demonstrated in Figure 3A, ILPMDA could obtain the highest AUC score when γ = 0.2. Thus, more previous label information for the node should be preserved when updating its label information. In addition, we also applied the AUCs of 5-CV to evaluate α ∈ {0, 0.1, 0.2,,1} for selecting the best value of parameter α. According to the evaluation results shown in Figure 3B, ILPMDA could obtain the highest AUC score while α = 0.3. This finding shows that the weight assigned to score matrix *F*_*d*_ should be greater than that of score matrix *F*_*m*_. The reason for this may be related to the number of collected miRNAs being higher than the number of collected diseases. In conclusion, the parameters γ and α in ILPMDA were set as 0.2 and 0.3, respectively.

### Performance Comparison

**FIGURE 4:**
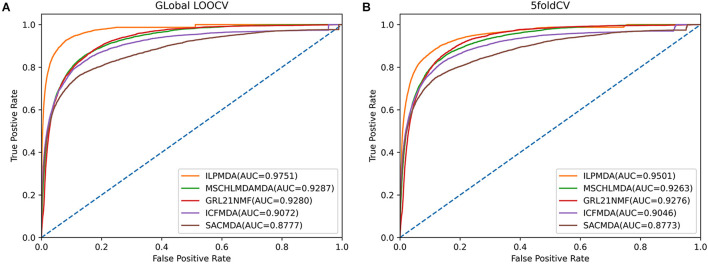
Performance comparisons of ILPMDA with MSCHLMDA, *GRL*_*2,1*_-NMF ICFMDA, and SACMDA in terms of AUC based on **(A)** global LOOCV and **(B)** 5-CV.

We first compared ILPMDA with several recent computational models [MSCHLMDA (Wu et al., 2020), *GRL*_2,1_-NMF (Gao et al., 2020), ICFMDA (Jiang Y. et al., 2018), and SACMDA (Shao et al., 2018)] to demonstrate its superior performance *via* the global LOOCV and 5-CV methods. Here, multi-similarity-based combinative hypergraph learning for predicting miRNA–disease association (MSCHLMDA) applied the KNN and k-means algorithms to establish different hypergraphs, which were combined to predict potential miRNA–disease associations. *GRL*_2,1_−*NMF*utilized the Laplacian regularized *L*_*2,1*_-nonnegative matrix factorization method to observe unknown miRNA–disease associations; ICFMDA incorporated similarity matrices to improve the collaborative filtering method for predicting more newer miRNA–disease pairs; and SACMDA utilized disease and miRNA information to construct a heterogeneous graph and used short acyclic connections in the heterogeneous graph to infer miRNA–disease associations. As illustrated in Figures 4A,B, ILPMDA achieved AUCs of 0.9751 and 0.9501 *via* global LOOCV and 5-CV, respectively. Both ranked the highest when compared with MSCHLMDA, *GRL*_2,1_-NMDA, ICFMDA, and SACMDA, which achieved AUCs of 0.9287, 0.9280, 0.9072, and 0.8777 in global LOOCV and 0.9263, 0.9276, 0.9046, and 0.8773 in 5-CV, respectively.

In addition, we compared ILPMDA with several LP-based models [MCLPMDA (Yu et al., 2019), HLPMDA (Chen et al., 2018b), and MDLPMDA (Qu et al., 2019)] to evaluate its prediction ability in the frameworks of global LOOCV and 5-CV. Here, MCLPMDA applied the matrix completion method to deal with similarities. Then, label propagation was applied to novel miRNA and disease similarity and association matrices to observe disease-related miRNAs. HLPMDA applied the heterogeneous label propagation algorithm on a multi-network of miRNAs, lncRNAs, and diseases to predict unobserved miRNA–disease interactions. MDLPMDA utilized the matrix decomposition method to obtain a novel association matrix with less noise and then applied the label propagation method to common miRNA and disease similarity matrices and the novel association matrix to infer unverified miRNA–disease associations. As illustrated in Table 1, MCLPMDA, HLPMDA, MDLPMDA, and ILPMDA achieved AUCs of 0.9320, 0.9218, 0.9211, and 0.9501, respectively, *via* the 5-CV method. By means of the global LOOCV method, MCLPMDA, HLPMDA, MDLPMDA, and ILPMDA achieved AUCs of 0.9410, 0.9232, 0.9222, and 0.9751, respectively. According to the above analysis, the prediction performance of ILPMDA is greater than that of previous computational models.

**TABLE 1:** Comparisons between ILPMDA and LP-based models.

**Models**	**AUC of 5-CV**	**AUC of global LOOCV**
MCLPMDA	0.9320	0.9410
HLPMDA	0.9218	0.9232
MDLPMDA	0.9211	0.9222
ILPMDA	0.9501	0.9751

**FIGURE 5:**
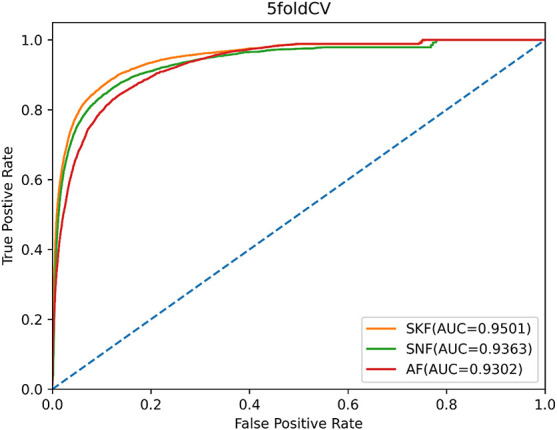
The ROC curves of SKF, SNF, and AF.

Moreover, we compared SKF with similarity network fusion (SNF) (Chen et al., 2019b) and average fusion to verify the superiority of SKF for integrating biology data. SNF fuses multiple pieces of complementary data to obtain integrated information, and the AF integrates different data by averaging multiple similarity matrices. In order to make a fair comparison, we used SNF and AF to replace SKF to process similarity data, while the remainder of the model remained unchanged. We also used the AUC values of 5-CV to evaluate the data integration capability of SKF, SNF, and AF. As shown in Figure 5, SKF, SNF, and AF obtained AUCs of 0.9501, 0.9364, and 0.9302, respectively. These findings demonstrate that SKF performs better than SNF and AF in terms of integrating different similarity information.

**FIGURE 6:**
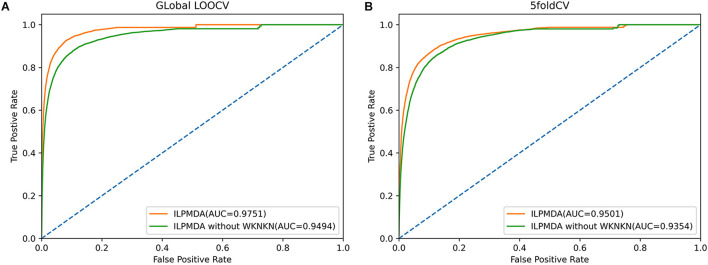
The ROC curves of ILPMDA and ILPMDA without WKNKN: **(A)** global LOOCV **(B)** 5-CV.

Furthermore, we also investigated the effect of the WKNKN algorithm for known miRNA–disease associations on model performance. We implemented two methods of global LOOCV and 5-CV, and then plotted the ROC curves, as shown in Figure 6. In ILPMDA, WKNKN considers the sparsity of the original association matrix, thereby improving the prediction performance of the model. By contrast, ILPMDA without WKNKN disregards the sparsity of the association data; thus, the predictive performance is also reduced. Based on the results, the AUC values of ILPMDA based on global LOOCV and 5-CV were 0.9751 and 0.9501, respectively. On the other hand, the AUC values of ILPMDA without WKNKN based on global LOOCV and 5-CV were 0.9494 and 0.9354, respectively. It is apparent that ILPMDA with WKNKN has higher AUC values compared to that without WKNKN.

### Case Studies

To demonstrate the prediction ability of ILPMDA, we implemented two common human diseases (colon neoplasms and prostate neoplasms) to perform a kind of case study. For a random disease, known associations of whole diseases in the HMDD v2.0 database were considered as training samples, while unknown associations were treated as candidate samples. We ranked the predicted association score of the candidate samples after performing ILPMDA; then, the top 50 candidate associations with the specific disease were selected and confirmed by the miR2Disease and dbDEMC v2.0 databases (Jiang et al., 2009; Yang et al., 2016). After we compared the information of the HMDD v2.0 database with that of the miR2Ddisease and dbDEMC databases, we found that 232 of the 5,430 verified associations in the HMDD v2.0 database also appeared in the miR2Disease database; meanwhile, 546 of the 5,430 verified associations in the HMDD v2.0 database also appeared in the dbDEMC v2.0 database. Furthermore, because only candidate samples for the specific disease were ranked and verified, the prediction list had no overlapping miRNAs in the training samples.

Colon neoplasms is acknowledged as the third gastrointestinal disease in the medical field (Torre et al., 2015; Bao et al., 2017). In addition, several potential miRNA–colon neoplasm connections have been observed in previous experiments (Hiroko et al., 2014; Rotelli et al., 2015), including miR-17, miR-21, and miR-31. These studies have shown that miRNAs can be utilized as key biomarkers for colon neoplasms. Hence, observing miRNA–colon neoplasm interactions can contribute to the diagnosis and treatment of colon neoplasms. After we ranked the prediction results of our model based on prediction score, 48 of the top 50 miRNAs were confirmed to be related to colon neoplasms according to the miR2Disease and dbDEMC v2.0 databases (Table 2).

**TABLE 2:** The top 50 potential miRNAs associated with colon neoplasms.

**miRNA**	**Evidence**	**miRNA**	**Evidence**
hsa-mir-145	m; d	hsa-mir-375	d
hsa-mir-126	m; d	hsa-mir-137	m; d
hsa-mir-17	m; d	hsa-mir-10b	m; d
hsa-mir-106a	m; d	hsa-mir-92a	d
hsa-mir-143	m; d	hsa-mir-196a	m; d
hsa-mir-155	m; d	hsa-mir-34a	m; d
hsa-mir-21	m; d	hsa-mir-223	m; d
hsa-mir-107	m; d	hsa-mir-19a	m; d
hsa-mir-200c	m; d	hsa-let-7f	m
hsa-mir-16	Unconfirmed	hsa-mir-630	d
hsa-mir-31	m; d	hsa-let-7e	d
hsa-mir-20a	m; d	hsa-let-7i	d
hsa-mir-200b	d	hsa-mir-622	d
hsa-mir-29a	m; d	hsa-mir-19b	m; d
hsa-mir-18a	m; d	hsa-mir-15a	d
hsa-let-7a	m; d	hsa-mir-192	m; d
hsa-mir-221	m; d	hsa-let-7c	d
hsa-mir-486	d	hsa-mir-629	d
hsa-mir-125b	d	hsa-mir-199a	Unconfirmed
hsa-mir-133b	m; d	hsa-mir-148a	d
hsa-let-7b	m; d	hsa-mir-9	m; d
hsa-mir-125a	m; d	hsa-mir-200a	d
hsa-mir-141	m; d	hsa-let-7g	m; d
hsa-mir-146a	d	hsa-mir-101	d
hsa-mir-142	d	hsa-mir-15b	m; d

*M, miR2Disease database; d, dbDEMC v2.0 database.*

Prostate neoplasms is regarded as the disease with the highest incidence rate among men; furthermore, these have been observed to associate with a part of some miRNAs in clinical experiments (Goto et al., 2015). For example, the expression of miR-183 in prostate cells and tissues is significantly higher than that in corresponding normal prostate cells and tissues. In conclusion, prostate neoplasms can be treated by inhibiting miR-183 expression (Ueno et al., 2013). After we ranked the prediction results of ILPMDA according to the prediction score, 46 of the top 50 miRNAs were confirmed to be associated with prostate neoplasms through the miR2Disease and dbDEMC v2.0 databases (Table 3).

**TABLE 3:** The top 50 potential miRNAs associated with prostate neoplasms.

**miRNA**	**Evidence**	**miRNA**	**Evidence**
hsa-mir-145	m; d	hsa-mir-146a	m
hsa-mir-125b	m; d	hsa-let-7c	m; d
hsa-mir-99a	m; d	hsa-mir-199a	m; d
hsa-mir-574	Unconfirmed	hsa-mir-34c	d
hsa-mir-183	m; d	hsa-mir-194	d
hsa-mir-141	m; d	hsa-let-7d	m; d
hsa-mir-100	m; d	hsa-mir-34a	m; d
hsa-mir-200c	d	hsa-mir-642a	Unconfirmed
hsa-mir-21	m; d	hsa-mir-92a	d
hsa-mir-200a	d	hsa-mir-629	d
hsa-mir-375	m; d	hsa-mir-155	d
hsa-mir-203	d	hsa-let-7a	m; d
hsa-mir-20a	m; d	hsa-mir-133b	d
hsa-mir-135b	Unconfirmed	hsa-mir-130b	d
hsa-mir-146b	d	hsa-mir-429	Unconfirmed
hsa-mir-708	d	hsa-mir-378a	d
hsa-mir-17	m; d	hsa-mir-29c	d
hsa-mir-143	m; d	hsa-mir-204	d
hsa-mir-205	m; d	hsa-mir-148a	m; d
hsa-mir-182	m; d	hsa-mir-96	m; d
hsa-mir-200b	d	hsa-mir-193b	d
hsa-mir-99b	m; d	hsa-mir-1	d
hsa-let-7b	m; d	hsa-mir-27a	m; d
hsa-mir-126	m; d	hsa-mir-218	m; d
hsa-mir-193a	d	hsa-mir-486	d

*M, miR2Disease database; d, dbDEMC v2.0 database.*

Next, we carried out another case study on breast neoplasms to illustrate the applicability of ILPMDA to new diseases. Breast neoplasms are often seen as a common disease in females, which have great negative effects on women’s health. Several miRNAs associated with breast neoplasms have been found by biological experiments in previous years (Fu et al., 2011). For example, the downregulation of miRNA-140 promoted cancer stem cell formation in basal-like early stage breast cancer (Li et al., 2014). In this case study, we deleted all miRNA–breast neoplasms association information from the HMDD v2.0 database, so breast neoplasms would be considered as a new disease without related miRNAs. We also used dbDEMC v2.0 and miR2Disease databases to validate predicted miRNAs related to breast neoplasms that were acquired by operating ILPMDA model. As shown in Table 4, **48** out of top 50 ranked miRNAs were verified by dbDEMC v2.0 database and miR2Disease database. Consequently, ILPMDA could be implemented to observe unverified miRNA–new disease associations.

**TABLE 4:** The top 50 potential miRNAs associated with breast neoplasms.

**miRNA**	**Evidence**	**miRNA**	**Evidence**
hsa-mir-1245a	d	hsa-mir-516a	d
hsa-mir-1245b	d	hsa-mir-103b	d
hsa-mir-1323	d	hsa-mir-320b	d
hsa-mir-1469	d	hsa-mir-200	Unconfirmed
hsa-mir-1471	d	hsa-mir-1915	d
hsa-mir-181	Unconfirmed	hsa-mir-376c	d
hsa-mir-2355	d	hsa-mir-526a	d
hsa-mir-298	d	hsa-mir-515	d
hsa-mir-299	d	hsa-mir-26a	m; d
hsa-mir-3130	d	hsa-mir-146b	m; d
hsa-mir-3186	d	hsa-mir-625	d
hsa-mir-411	d	hsa-mir-301b	m; d
hsa-mir-4257	d	hsa-mir-450b	d
hsa-mir-4306	d	hsa-mir-139	d
hsa-mir-632	d	hsa-mir-129	d
hsa-mir-718	d	hsa-mir-506	d
hsa-mir-874	d	hsa-mir-26b	d
hsa-mir-922	d	hsa-mir-510	m; d
hsa-mir-493	d	hsa-mir-1258	d
hsa-mir-147a	d	hsa-mir-128	d
hsa-mir-202	m; d	hsa-mir-513a	d
hsa-mir-320d	d	hsa-mir-519d	d
hsa-mir-320e	d	hsa-mir-193a	d
hsa-mir-450a	d	hsa-mir-215	d
hsa-mir-516b	d	hsa-mir-488	d

*m, miR2Disease database; d, dbDEMC v2.0 database.*

According to the above analysis, the case studies of colon neoplasms and prostate neoplasms further demonstrate the utility of our model in predicting unknown miRNA–disease associations.

## Discussion

In this paper, we introduced a novel method, i.e., ILPMDA, in which we employed an improved label propagation algorithm to predict possible miRNA–disease associations. In this model, SKF was employed to integrate different disease and miRNA similarities. After fusion, the final disease and miRNA similarity networks were obtained, and WKNKN was applied to reduce the sparsity of the known miRNA–disease association matrix. We then applied the KNN algorithm to sort k-nearest neighbors for entity nodes on two types of similarity networks, thereby ensuring that weighted disease and miRNA networks could be constructed for appropriately implementing label propagation. In addition, we implemented the bidirectional label propagation algorithm on the weighted disease and miRNA similarity networks to generate different association score matrices, which were integrated to acquire the ultimate prediction score of each miRNA–disease pair. In the framework of global LOOCV and 5-CV, the AUCs of our model were 0.9751 and 0.9501, respectively. Based on these results, the performance of ILPMDA was superior to that of various previous prediction models. The case studies on colon neoplasms, prostate neoplasms, and breast neoplasms also confirmed the prediction ability of ILPMDA.

The following factors may contribute to the reliable performance of ILPMDA. First, the SKF algorithm was implemented to integrate various disease and miRNA similarities, which provide plentiful biological information for the experiment. In addition, the label propagation algorithm can be carried out to construct weighted disease and miRNA similarity matrices. Furthermore, the principle of bidirectional label propagation ensured that the labels of candidate entity nodes were steadily updated, which allowed us to obtain accurate experimental results.

However, there are certain limitations to ILPMDA. The data we utilized included verified miRNA–disease associations, miRNA similarity information, and disease similarity information, which may lead to the inclusion of noise and outliers. In addition, ILPMDA was only suitable for diseases and miRNAs that are hosted on the HMDD v2.0 database. Therefore, our model should be continuously optimized in the future to improve its performance.

## Data Availability Statement

The original contributions presented in the study are included in the article/supplementary material, further inquiries can be directed to the corresponding authors.

## Author Contributions

Y-TW developed the prediction method and designed the experiment. Y-TW and LL performed the experiment and wrote the manuscript. C-MJ processed the data. C-HZ and J-CN conceived and supervised the entire project and revised the manuscript. All authors contributed to the article and approved the manuscript.

## Conflict of Interest

The authors declare that the research was conducted in the absence of any commercial or financial relationships that could be construed as a potential conflict of interest.

## Publisher’s Note

All claims expressed in this article are solely those of the authors and do not necessarily represent those of their affiliated organizations, or those of the publisher, the editors and the reviewers. Any product that may be evaluated in this article, or claim that may be made by its manufacturer, is not guaranteed or endorsed by the publisher.
